# Home‐Based Combined Activity and Cognitive Intervention for Post‐Intensive Care Syndrome: A Pilot Randomised Controlled Trial

**DOI:** 10.1111/nicc.70548

**Published:** 2026-06-25

**Authors:** Polly W. C. Li, C. K. Tong, Peter C. K. Lai, Pauline Y. Ng, Sampson C. S. Chan, M. H. Ho, Doris S. F. Yu

**Affiliations:** ^1^ School of Nursing, LKS Faculty of Medicine The University of Hong Kong Hong Kong China; ^2^ Intensive Care Unit Tuen Mun Hospital, Hospital Authority of Hong Kong Hong Kong China; ^3^ Adult Intensive Care Unit Queen Mary Hospital, Hospital Authority of Hong Kong Hong Kong China; ^4^ Critical Care Medicine Unit, School of Clinical Medicine, LKS Faculty of Medicine The University of Hong Kong Hong Kong China; ^5^ Intensive Care Unit, Department of Medicine and Geriatrics Ruttonjee & Tang Shiu Kin Hospitals, Hospital Authority of Hong Kong Hong Kong China

**Keywords:** cognitive rehabilitation, combined exercise and cognitive intervention, home‐based rehabilitation, ICU survivors, post‐intensive care syndrome

## Abstract

**Background:**

ICU survivors frequently develop post‐intensive care syndrome (PICS), a cluster of persistent physical, cognitive and psychological impairments that substantially impair recovery and quality of life. Existing rehabilitation approaches are predominantly monomodal and exercise‐focused, yielding inconsistent outcomes and failing to address the multidimensional burden of PICS adequately.

**Aim:**

To evaluate the feasibility and preliminary efficacy of COMBAT‐ICU, a home‐based Combined Activity and Cognitive Intervention for ICU survivors at risk of PICS.

**Study Design:**

A parallel, three‐arm, assessor‐blinded pilot randomised controlled trial randomised 36 ICU survivors (1:1:1) to COMBAT‐ICU—an 8‐week blended program of progressive physical exercise and computerised cognitive training delivered via supervised home visits and online sessions—an exercise‐only group or an attention control group. The primary outcomes were feasibility (recruitment, retention and intervention adherence) and safety; secondary exploratory outcomes encompassed PICS severity, physical capacity, cognition, mental health and health‐related quality of life (HRQoL).

**Results:**

COMBAT‐ICU was feasible and safe (36 ICU survivors randomised), with no serious adverse events recorded, retention exceeding 82% at follow‐up and session adherence exceeding 90%. COMBAT‐ICU produced significantly greater reductions in PICS severity versus attention control at post‐intervention (*p* = 0.014, *d* = −0.50) and follow‐up (*p* = 0.043, *d* = −0.45). It also yielded clinically meaningful moderate‐to‐large effect sizes for walking endurance, global cognition, short‐term memory and HRQoL index scores compared with attention control and consistently outperformed exercise‐only across cognitive and HRQoL domains. Between‐group differences in anxiety and depressive symptoms were small across all active groups.

**Conclusions:**

COMBAT‐ICU is feasible and shows promising preliminary efficacy in mitigating PICS. Integrating cognitive and physical training within a home‐based blended delivery model may confer synergistic benefits beyond exercise alone, providing domain‐specific effect size estimates and a compelling rationale for definitive multicentre trials.

**Relevance to Clinical Practice:**

Multidomain home‐based rehabilitation is a viable post‐discharge strategy for ICU survivors. COMBAT‐ICU offers an evidence‐informed, scalable framework to enhance survivorship care, pending confirmation in larger, fully powered trials.

**Trial Registration:**

The trial was registered at ClinicalTrials.gov (NCT06117761)

## Impact Statements

- What is known about this topic
  ○ ICU survivors frequently experience persistent physical, cognitive and psychological impairments, yet access to structured post‐ICU rehabilitation is limited.
  ○ Exercise‐based post‐ICU programs show benefits for physical function but often have high attrition and only small or inconsistent between‐group effects.
  ○ Combined motor–cognitive interventions improve cognition and function in other populations, but have been little studied in ICU survivors.
- What this paper adds
  ○ A home‐based combined activity and cognitive program (COMBAT‐ICU) for ICU survivors at risk of PICS is feasible, with high retention and adherence using a blended home visit and online model.
  ○ COMBAT‐ICU yields small‐to‐medium effects across PICS severity, physical and cognitive outcomes and health‐related quality of life.
  ○ The trial provides effect size estimates and practical parameters to inform the design and powering of future definitive post‐ICU rehabilitation studies.

## 1 Introduction

As survival following critical illness has improved, attention has shifted from mortality to the long‐term functional recovery and health‐related quality of life (HRQoL) of intensive care unit (ICU) survivors. A substantial proportion develop post‐intensive care syndrome (PICS), characterised by persistent physical, cognitive and psychological impairments that can persist for months or even years [1], and limit activities of daily living, impede return to work and restrict social participation [2]. PICS is also associated with higher healthcare utilisation, readmission and mortality within the first year after discharge [3], and has been identified as a key research priority [2].

## 2 Background

The pathophysiology of PICS is multifaceted, but it is thought to arise in part from ‘learned non‐use’ secondary to prolonged immobilisation, deep sedation and sensorimotor deprivation during critical illness [1]. To counteract these detrimental mechanisms, guideline‐based care bundles, such as the ABCDEF bundle, advocate for early mobilisation, the minimisation of sedation, spontaneous breathing trials and psychological support [4]. However, while early mobilisation reliably improves short‐term physical outcomes within the ICU setting, meta‐analytic evidence indicates that these functional gains frequently dissipate within six months of discharge. This attenuation suggests that therapeutic interventions confined solely to the acute phase of illness are insufficient to sustain long‐term recovery [5].

Although various post‐ICU rehabilitation trials have been conducted, their outcomes remain largely equivocal. Physical rehabilitation alone exerts inconsistent effects on functional capacity, without reliably improving HRQoL or reducing mortality [6, 7]. Likewise, post‐ICU follow‐up clinics and enhanced services yield only modest psychological benefits, with negligible physical impact [8, 9]. Furthermore, transitional and home‐based exercise programs are frequently compromised by high attrition, poor adherence and inadequate training intensity, which limits their ability to accelerate recovery [10, 11]. Collectively, these findings underscore a critical therapeutic mismatch: despite widespread guidelines advocating for integrated care, predominantly monomodal, exercise‐focused interventions fall short of addressing the complex physical, cognitive and psychological dimensions of PICS.

To address these gaps, we developed COMBAT‐ICU, a home‐based program that integrates physical exercise with computer‐based cognitive training for ICU survivors at risk of PICS. By leveraging motor–cognitive synergy within a home setting, COMBAT‐ICU aims to reduce access barriers, support sustained participation and more directly target the multidimensional impairments of post‐critical illness recovery.

### 2.1 Aims and Objectives

#### 2.1.1 Aim

This study aimed to evaluate the feasibility and preliminary efficacy of COMBAT‐ICU among ICU survivors at risk of PICS.

#### 2.1.2 Objectives

1. Assess feasibility in terms of participant recruitment, retention and intervention adherence and evaluate safety through adverse event monitoring;
2. Examine the preliminary efficacy of COMBAT‐ICU compared with an exercise‐only program and an attention control on PICS severity, physical function, cognitive function, mental health and health‐related quality of life at post‐intervention and three‐month follow‐up;
3. Generate domain‐specific effect size estimates to inform the design and sample size calculation of a future definitive randomised controlled trial (RCT).

## 3 Methods

### 3.1 Design

This study was a parallel, three‐arm, assessor‐blinded pilot RCT reported in accordance with CONSORT guidelines.

### 3.2 Setting and Sample

Participants were prospectively recruited from the ICUs of three regional hospitals in Hong Kong. In Hong Kong's public hospital system, ICU survivors are routinely transferred to a general ward or high‐dependency step‐down unit for further clinical stabilisation before final hospital discharge, with direct ICU‐to‐community discharge being uncommon. Participants were therefore identified and approached during their step‐down ward stay prior to final hospital discharge. Eligibility criteria included adults aged 18 years or older who were admitted to the ICU for a minimum of four days, as prolonged admission significantly increases the risk of developing long‐standing PICS [12]. Additional inclusion criteria required that patients' final hospital discharge destination be their own home in the community (i.e., not transferred to a convalescent hospital, nursing home or other institutional care facility), be capable of performing basic activities of daily living independently prior to ICU admission, live with family members, have access to an internet‐enabled electronic device and possess the ability to walk for at least 10 m (assisted or unassisted) at the time of recruitment.

Patients were excluded if they had pre‐existing musculoskeletal injuries precluding exercise training, were already receiving structured outpatient pulmonary or cardiac rehabilitation, scored < 6/10 on the Abbreviated Mental Test at the time of recruitment or presented with clinically evident dementia or significant impairments resulting from acute neurological insults (such as traumatic brain injury, stroke or hypoxic brain injury) that would preclude adherence to the study protocol. Furthermore, individuals with a prolonged length of stay (defined as 28 days or more) in step‐down wards were excluded, as their long‐term impairment trajectories and rehabilitation needs are likely altered by the extended hospitalisation.

### 3.3 Study Interventions

#### 3.3.1 COMBAT‐ICU Group

Participants received an 8‐week home‐based combined exercise and cognitive intervention (3 sessions/week; 45 min exercise + 5‐min break + 30 min cognitive training). A blended platform combined supervised home visits, real‐time online sessions and unsupervised self‐practice, transitioning by functional level; family caregivers were involved to support home training. Full intervention details are provided in the TIDieR framework (Table 1).

**TABLE 1:** Description of the COMBAT‐ICU Intervention based on the TIDieR (Template for Intervention Description and Replication) checklist.

TIDieR item	Description for COMBAT‐ICU
1. Brief name	COMBAT‐ICU (A home‐based, combined exercise and cognitive intervention).
2. Why (rationale)	Designed to address the marked heterogeneity in physical and cognitive deficits among intensive care unit (ICU) survivors upon hospital discharge. The gradual blending of supervision aims to boost self‐efficacy, motivation and multidomain recovery.
3. What (materials)	Physical: Simple home‐based exercise equipment (elastic resistance bands, ankle/cuff weights), activity tracker (for resting heart rate monitoring). Cognitive: Computer or tablet with the scientifically validated CogniFit platform. Educational/Support: Standardised functional assessment protocol (appendix 3) and exercise videos to guide self‐practice.
4. What (procedures)	Total session duration: 80 min Exercise Training (45 min): 10 min of warm‐up/cool‐down (flexibility/stretching) and 35 min of core exercise targeting mobility, balance, strength and endurance across four individualised functional levels: ○ Level 1 (most impaired): Static balance, guarded gait, bodyweight resistance (seated toe raises, guarded standing), short walking bouts with rest breaks ○ Level 2: Dynamic balance, gait variability drills (direction changes, accelerated gait), light resistance bands, progressive walking intervals ○ Level 3: Advanced dynamic balance, moderate resistance training (4‐limb large muscle groups with bands/cuff weights), continuous walking with chair/stepping exercises ○ Level 4 (least impaired): Primarily endurance and resistance training with higher loads/lower repetitions for muscle power; varied exercise modalities per participant preference Break (5 min): Rest transition. Cognitive Training (30 min): Conducted immediately post‐exercise. Includes 4 cross‐modality brain‐stimulating games (targeting attention, memory, executive function) and 1 ongoing cognitive assessment Integration: Interveners assess the home environment to provide individualised advice on integrating exercises into daily routines.
5. Who provided	Delivered by trained interveners with rehabilitation or sports science backgrounds. Family caregivers are explicitly involved in the intervention to facilitate and support home‐based training.
6. How (mode of delivery)	A blended delivery approach featuring: In‐person supervised home visits. Real‐time, remote online supervision (via WhatsApp video call or Zoom). Unsupervised, video‐guided self‐practice.
7. Where (setting)	The participant's own home environment.
8. When and how much	Duration: 8 weeks. Frequency: 3 sessions per week (80 min/session). Target intensity: Resting heart rate + 20–30 beats/min and a Rate of Perceived Exertion (RPE) of 12–14 (‘somewhat hard’).
9. Tailoring	Physical: Participants are categorised into Functional Levels 1–4 weekly. Lower levels (1 and 2) focus on bodyweight balance/mobility and short walking bouts. Higher levels (3 and 4) focus on endurance/resistance using bands/weights. Delivery mode escalation: *Level 1 and 2 participants:* Receive 100% in‐person home visits until functional progression to Level 3–4 is confirmed by weekly performance assessment, at which point delivery transitions to the blended format. *Level 3 and 4 participants:* Shift gradually from Induction (Weeks 1–2: 2 home visits, 1 online) ➔ Maintenance (Weeks 3–6: 1 home visit, 2 online) ➔ Consolidation (Weeks 7–8: 2 online, 1 self‐practice). Cognitive: Baseline 20‐min assessment tailors initial difficulty. CogniFit software automatically scales complexity in Weeks 1–4. In Weeks 5–8, participants self‐select games based on interest.
10. Modifications	Not applicable
11. How well (planned)	Monitoring/fidelity: Exercise progression is strictly guided by objective (activity tracker HR) and subjective (RPE 6–20 scale) metrics. Cognitive progression is automatically tracked via the CogniFit software's built‐in assessment tasks.
12. How well (actual)	Delivery and adherence: The intervention was delivered with high fidelity and acceptance. The COMBAT‐ICU group (*n* = 13) achieved a mean session attendance rate of 91.3% (comparable to 89.9% in the exercise‐only group). Retention and attrition: The COMBAT‐ICU group achieved a 100% follow‐up assessment completion rate at both T1 and T2. The observed attrition in the control/exercise‐only groups was attributable to the inability to contact participants at scheduled assessment time points.

Home visits were conducted by research assistants with rehabilitation science backgrounds who received structured training from the research team in the standardised exercise protocol, functional performance assessment, safety monitoring and motivational communication techniques. Visit scheduling was coordinated with participants and their family caregivers via telephone 1–2 days in advance. During visits, the research assistant guided participants through the tailored exercise protocol, monitored vital signs (resting heart rate, oxygen saturation, blood pressure), assessed safety and tolerance using the Rate of Perceived Exertion scale and provided real‐time feedback on technique and progression. Family caregivers were present during visits and were trained to support participants' self‐practice between sessions.

Exercise training comprised three progressive phases. Weeks 1–2 (Induction): two supervised home visits and one online session per week, establishing exercise familiarity and safety monitoring. Exercise targeted four domains (mobility, balance, strength, endurance) across four individualised performance levels [13]. Weeks 3–6 (Maintenance): delivery was adjusted by functional level—participants at Levels 3–4 received one home visit and two online sessions per week; those at Levels 1–2 continued to receive three home visit sessions per week until functional progression to Level 3–4, at which point delivery transitioned to the blended format. Weeks 7–8 (Consolidation): two online sessions and one self‐directed session per week. Intensity was progressive (resting heart rate: +20–30 beats/min; Rate of Perceived Exertion (RPE): 12–14) using home‐compatible equipment (elastic bands, ankle weights), with progression monitored via activity trackers, RPE and symptom review.

Cognitive training was delivered through the CogniFit platform, a validated computerised cognitive training program with demonstrated reliability across multiple clinical populations [14]. Baseline assessment (15 tasks, ~20 min) established individual difficulty levels. Each session comprised four cross‐modality brain games targeting attention, memory and executive function, plus one cognitive assessment task. During Weeks 1–4, game complexity adjusted automatically; from Week 5, participants selected games by interest while maintaining task mastery.

#### 3.3.2 Exercise‐Only Group

Participants received an 8‐week, home‐based exercise intervention. The implementation protocol was identical to the exercise component of the COMBAT‐ICU intervention, with 3 sessions per week and 45 min per session. They did not receive any structured cognitive training.

#### 3.3.3 Attention Control Group

Participants received biweekly telephone calls (10–15 min) from a trained research nurse, providing general health information and brief non‐directive counselling. This attention control condition was designed to match the frequency of structured researcher contact inherent in the active intervention groups, thereby isolating the specific therapeutic effects of physical and cognitive training from the non‐specific effects of social interaction and researcher attention on patient‐reported outcomes. They continued receiving standard care (discharge planning, medication management, outpatient medical follow‐up) but no structured physical, pulmonary or cardiac rehabilitation.

### 3.4 Data Collection Procedures

Prior to transfer from the ICU to step‐down wards, clinical staff informed patients and family members about the study. Upon hospital discharge, a trained research assistant screened patients for eligibility and provided written study information supplemented with verbal clarification. Eligible patients provided written informed consent, after which baseline data were collected. Participants were then randomly assigned to one of three groups: COMBAT‐ICU, exercise‐only or attention control.

Stratified block randomisation by study site ensured balanced group allocation across the three participating hospitals, accounting for variations in patients' socio‐economic status and clinical practice. Participants were allocated in a 1:1:1 ratio using a computer‐generated randomisation sequence with variable‐sized blocks (sizes 3 and 6) specific to each site stratum. A biostatistician independent of the research team generated site‐specific randomisation lists prior to study commencement. Sequentially numbered, opaque, sealed envelopes corresponding to each site‐specific list were prepared centrally, packaged separately for each site and held by the local site coordinator who was not involved in participant assessment or intervention delivery. Upon eligibility confirmation and consent, the local coordinator opened the next envelope in sequence for that site, maintaining allocation concealment independently at each centre. To minimise performance and detection biases, the clinical care team (physicians, nurses and allied health personnel) and the post‐intervention data collector were blinded to group allocation throughout the study period.

### 3.5 Data Collection Tools

Feasibility was evaluated using recruitment, retention and adherence metrics. Recruitment feasibility was described using referral, screening and enrollment numbers and reasons for ineligibility or refusal. Retention was assessed via completion rates of T1 and T2 assessments and reasons for loss to follow‐up. Intervention adherence was quantified as the percentage of completed versus scheduled training sessions for each intervention group. Safety was monitored throughout the intervention period. All adverse events and serious adverse events (defined as any unintended medical occurrence resulting in hospitalisation, significant disability or requiring medical intervention) were recorded by the research assistants at each session and reported to the principal investigator. Participants were withdrawn from the intervention if a serious adverse event was deemed related to the study activities.

Several performance‐based and patient‐reported measures were used to capture changes at baseline (T0), immediate post‐intervention (T1) and 3 months post‐intervention (T2). The severity of patient‐reported PICS, measured using the 18‐item Post‐Intensive Care Syndrome Questionnaire (PICSQ) [15], which comprehensively evaluates the physical, cognitive and mental dimensions of the syndrome. Secondary outcomes encompassed measures of physical function, cognitive function, mental health and HRQoL. Physical function was quantified using the 6‐Minute Walk Test (6MWT) [16] to assess aerobic capacity and walking endurance in meters, alongside the Short Physical Performance Battery (SPPB) [17], which evaluates standing balance, usual gait speed and repeated chair stands. Cognitive function was systematically evaluated across multiple domains: global cognition was measured using the Montreal Cognitive Assessment (MoCA) [18]; attention, processing speed and executive function were assessed via the Colour Trails Test (CTT‐1 and CTT‐2) [19]; and short‐term and working memory were evaluated using the Digit Span test (forward and backward configurations) (DST‐F and DST‐B) [20]. Mental health parameters were captured using the Patient Health Questionnaire‐9 (PHQ‐9) [21] to quantify depressive symptomology and the Generalized Anxiety Disorder Scale‐7 (GAD‐7) [22] for anxiety symptoms. Finally, HRQoL was measured utilising the EQ‐5D‐5L [23], consisting of a five‐dimension descriptive system—covering mobility, self‐care, usual activities, pain or discomfort and anxiety or depression—and a visual analogue scale (VAS) ranging from 0 to 100.

### 3.6 Data Analysis

Because this was a feasibility study, no formal power analysis was conducted for efficacy outcomes. The sample size of 12 participants per arm (total *n* = 36) was selected to assess feasibility outcomes to inform the design of a future definitive trial [24].

Baseline demographic and clinical characteristics were compared across the three study arms using the *χ* ^2^ test for categorical variables and one‐way ANOVA or the Kruskal–Wallis test for continuous variables. To explore between‐group differential changes across the three arms over time, generalised estimating equation (GEE) modelling was applied, adjusting for pre‐specified baseline covariates, including age, sex, prior mental health history, disease severity and negative ICU experience (defined as the presence of distressing or frightening memories of the index ICU stay) [25]. Effect sizes were determined by calculating Cohen's d based on mean between‐group differences in score changes from baseline to each specific follow‐up time point and were interpreted according to conventional ranges: small (0.20–0.49), medium (0.50–0.79) and large (≥ 0.80). All statistical analyses strictly adhered to the intention‐to‐treat principle and were conducted using IBM SPSS version 30.

### 3.7 Ethical and Institutional Approvals

The study received ethical approval (reference number: 6 June 2023 (UW‐23‐271) and 28 Nov 2023 (CIRB‐2023‐087‐2)) from the institutional review boards of all participating hospitals. All participants provided written informed consent.

## 4 Results

### 4.1 Feasibility: Participant Recruitment, Retention and Adherence

**FIGURE 1:**
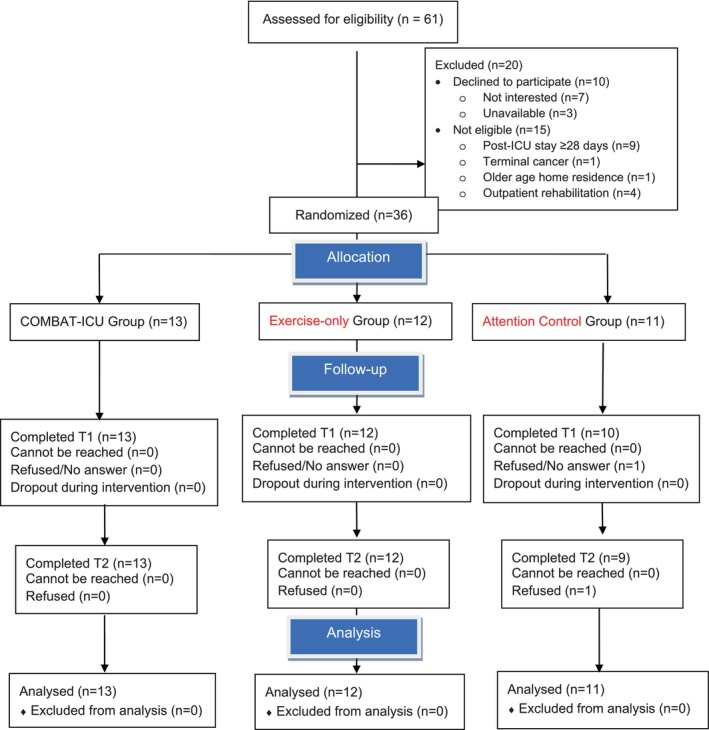
The CONSORT diagram.

Of 80 referred ICU patients, 19 were unreachable post‐discharge and 15 did not meet the eligibility criteria; 10 declined participation (*n* = 7 not interested; *n* = 3 unavailable). A total of 36 participants were ultimately enrolled and randomised, with 13 allocated to COMBAT‐ICU, 12 to exercise‐only and 11 to the attention control group. Figure 1 shows the CONSORT diagram.

The COMBAT‐ICU group achieved 100% completion at T1 and T2. The exercise‐only group completed T1 assessment without loss (*n* = 12/12) but achieved 92% at T2 (*n* = 11/12; one participant unable to be reached). The attention control group completed T1 assessment with 91% retention (*n* = 10/11; one participant unavailable) and T2 assessment with 82% retention (*n* = 9/11; one participant unreachable). Session‐level adherence was high in both active intervention groups (Table 2). The COMBAT‐ICU group completed 285 of 312 scheduled sessions (adherence rate: 91.3%), and the exercise‐only group completed 259 of 288 scheduled sessions (adherence rate: 89.9%). The most common reasons for missed sessions were competing commitments/illness. No participants discontinued the intervention.

**TABLE 2:** Feasibility, adherence and safety outcomes of the COMBAT‐ICU, exercise‐only and attention control groups.

Feasibility parameter	COMBAT‐ICU (*n* = 13)	Exercise‐only (*n* = 12)	Attention control (*n* = 11)
Recruitment			
Enrolled/referred, *n* (%)	36/80 (45%)	—	—
Retention			
T1 completion, *n* (%)	13/13 (100%)	12/12 (100%)	10/11 (91%)
T2 completion, *n* (%)	13/13 (100%)	11/12 (92%)	9/11 (82%)
Adherence			
Sessions completed, *n*/*N*	285/312^a^	259/288^a^	—
Session adherence rate, %	91.3%	89.9%	—
Safety			
Serious adverse events, *n*	0	0	0
Minor adverse events, *n*			—
Muscle soreness	7	8	
Joint pain	0	0	
Muscle strain	0	0	
Muscle sprain	0	0	

^a^ Total scheduled sessions = 3 sessions/week × 8 weeks × *n* participants = 312 sessions for COMBAT‐ICU (13 × 24) and 288 sessions for exercise‐only (12 × 24).

### 4.2 Safety

No serious adverse events attributable to the intervention were recorded during the trial. Exercise‐associated muscle soreness, an anticipated physiological response to progressive training, was noted among some participants but not classified as an adverse event per protocol. These findings support the safety and tolerability of progressive home‐based exercise and cognitive training in this clinically vulnerable population.

### 4.3 Sample Characteristics

The mean age was 65.1 years (SD 14.6); approximately half were male (52.8%), most were married (83.3%) and living with others (94.4%). At baseline, participants demonstrated moderate functional impairment (mean SPPB: 8.8 out of 12, SD 1.89; mean 6MWT: 372.8 m, SD 106.87), largely intact global cognition (mean MoCA: 25.6, SD 3.52) and mild‐to‐moderate symptoms of anxiety (mean GAD‐7: 3.7, SD 2.54) and depression (mean PHQ‐9: 5.8, SD 2.59). Groups were broadly comparable at baseline across socio‐demographic, clinical and outcome variables (Table 3).

**TABLE 3:** Participant characteristics at baseline.

	All (*n* = 36) frequency (%)	COMBAT‐ICU group (*n* = 13) frequency (%)	Exercise group (*n* = 12) frequency (%)	Control group (*n* = 11) frequency (%)
Socio‐demographic characteristics				
Age (mean ± SD)	65.12 ± 14.56	63.90 ± 15.32	65.44 ± 14.53	69.17 ± 13.69
Gender				
Male	17 (47.2)	7 (53.8)	4 (33.3)	6 (54.5)
Female	19 (52.8)	6 (46.2)	8 (66.7)	5 (45.5)
Marital status				
Married	30 (83.3)	10 (76.9)	11 (91.7)	9 (81.8)
Single/divorced/widowed	6 (16.7)	3 (23.1)	1 (8.3)	2 (18.2)
Education level				
Nil/Primary	12 (33.3)	4 (30.8)	4 (33.3)	4 (36.4)
Secondary 1–3	6 (16.7)	1 (7.7)	3 (25.0)	2 (18.2)
Secondary 4–7	13 (36.1)	4 (30.8)	4 (33.3)	5 (45.5)
Tertiary or above	5 (13.9)	4 (30.8)	1 (8.3)	0 (0.0)
Living arrangement				
Living with others	34 (94.4)	12 (92.3)	11 (91.7)	11 (100.0)
Living alone	2 (5.6)	1 (7.7)	1 (8.3)	0 (0.0)
Household income (HKD)^a^				
None/<$10 000	20 (55.6)	4 (30.8)	6 (50.0)	7 (63.6)
$10 001–$20 000	2 (5.6)	0 (0.0)	1 (8.3)	2 (18.2)
> $200 00	14 (38.9)	9 (69.2)	5 (41.7)	2 (18.2)
Occupation				
Unemployed/retired/home duties	25 (69.4)	7 (53.8)	9 (75.0)	9 (81.8)
Employed	11 (30.6)	6 (46.2)	3 (25.0)	2 (18.2)
Clinical characteristics				
Smoking history				
Never smoked	27 (75.0)	8 (61.5)	8 (66.7)	11 (100)
Smoking	4 (11.1)	1 (7.7)	3 (25.0)	0 (0.0)
Previously smoked	5 (13.9)	4 (30.8)	1 (8.3)	0 (0.0)
*ICU admission diagnosis*				
*Cardiovascular*	***15 (41.7)***	***6 (46.2)***	***5 (41.7)***	***4 (36.4)***
*Pulmonary*	***8 (22.2)***	***3 (23.1)***	***3 (25.0)***	***2 (18.2)***
*Kidney*	***3 (8.3)***	***1 (7.7)***	***0 (0)***	***1 (9.1)***
*Gastrointestinal*	***2 (5.6)***	***0 (0)***	***1 (8.3)***	***1 (9.1)***
*Surgical*	***8 (22.2)***	***3 (23.1)***	***2 (16.7)***	***3 (27.3)***
*APACHE II score (mean, SD)*	***18.61 (8.43)***	***18.41 (7.97)***	***18.13 (6.24)***	***19.03 (6.58)***
*Mechanical ventilation*	***18 (50.0)***	***6 (46.2)***	***6 (50.0)***	***6 (54.5)***
*Length of stay (mean, SD)*				
*Hospital*	***26.0 (14.8)***	***27.1 (15.8)***	***24.0 (13.6)***	***26.6 (13.9)***
*ICU*	***11.1 (8.0)***	***11.4 (8.9)***	***9.6 (7.4)***	***12.3 (7.8)***
*Mobility before ICU admission*				
*Walk without aid*	***31 (86.1)***	***11 (84.6)***	***11 (91.7)***	***9 (81.8)***
*Walk with an aid*	***5 (13.9)***	***2 (15.4)***	***1 (8.3)***	***2 (18.2)***
*Charlson Comorbidity Index score (mean, SD)*	***2.21 (1.25)***	***2.12 (1.31)***	***2.20 (1.28)***	***2.22 (1.27)***
Outcome measures (mean, SD)				
PICSQ	25.72 (3.92)	25.54 (3.73)	25.75 (4.59)	25.91 (13.70)
SPPB	8.75 (1.89)	8.69 (2.32)	8.75 (1.55)	8.82 (1.83)
6MWT	372.78 (106.87)	365.38 (112.52)	376.67 (104.39)	377.27 (112.61)
MoCA	25.56 (3.52)	25.23 (2.62)	25.83 (4.95)	25.64 (2.80)
CTT2 time	120.32 (45.10)	121.22 (40.80)	115.12 (48.27)	124.92 (49.99)
CTT1 time	59.36 (20.39)	58.52 (14.58)	60.22 (17.57)	59.41 (29.29)
DST (F)	7.36 (0.90)	7.31 (1.86)	7.50 (1.00)	7.27 (0.91)
DST (B)	4.36 (0.96)	4.46 (0.88)	4.33 (1.37)	4.27 (0.47)
GAD‐7	3.72 (2.54)	3.77 (2.00)	3.67 (3.14)	3.73 (2.61)
PHQ‐9	5.83 (2.59)	5.77 (1.30)	5.75 (3.96)	6.00 (2.05)
EQ‐5D‐5L index	0.61 (0.13)	0.60 (0.12)	0.63 (0.11)	0.60 (0.17)
EQ‐5D‐5L VAS	60.56 (12.41)	60.00 (12.75)	59.58 (14.99)	62.27 (9.58)

Abbreviations: 6MWT, 6‐min walk test; CCT, Colour Trail Test; DST (B), Digit Span Test (forward); DST, Digit Span Test (backward); EQ‐5D‐5L VAS, EuroQol 5‐Dimension 5‐Level (visual analogue scale); GAD‐7, Generalised Anxiety Disorder 7‐item scale; MoCA, Montreal Cognitive Assessment; n, number; PHQ‐9, Patient Health Questionnaire‐9; PICSQ, Post‐Intensive Care Syndrome questionnaire; SD, standard deviation; SPPB, Short Physical Performance Battery.

^a^ 1USD = 7.8 HKD.

### 4.4 Exploratory Outcomes

**FIGURE 2:**
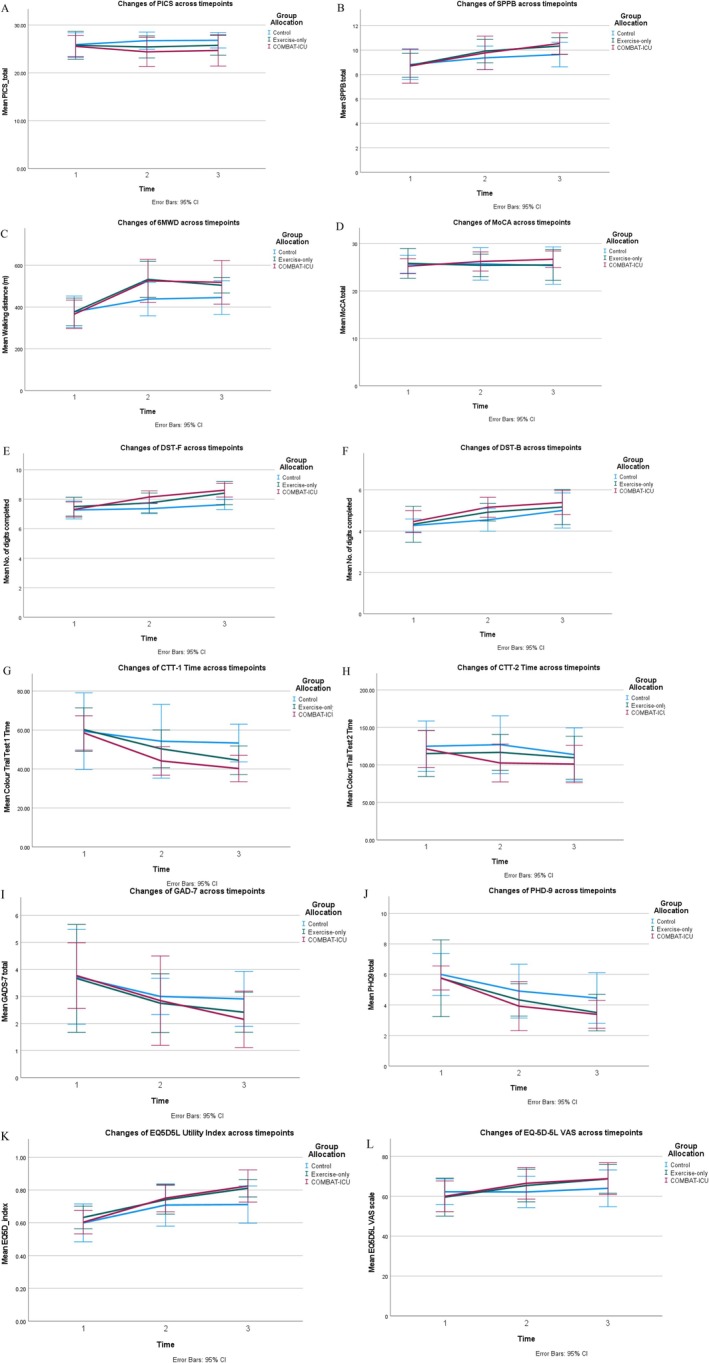
Changes in outcomes over time. (A) Post‐intensive care syndrome symptoms measured by the Post‐Intensive Care Syndrome Questionnaire (PICSQ); (B) Aerobic capacity and walking endurance measured by the 6‐Minute Walk Test (6MWT); (C) Physical function measured by the Short Physical Performance Battery (SPPB); (D) Global cognition measured by the Montreal Cognitive Assessment (MoCA); (E, F) Memory measured by the Digit span test (forward and backward); (G, H) Executive function measured by the Colour Trails Test; (I) Anxiety symptoms measured by the Generalised Anxiety Disorder Scale‐7 (GAD‐7); (J) Depressive symptoms measured by the Patient Health Questionnaire‐9 (PHQ‐9); (K, L) Health‐related quality of life measured by the EuroQol 5‐Dimension 5‐Level.

Full between‐group comparisons across all secondary outcomes are presented in Table 4 and Figure 2. Given the pilot nature of this study and the small sample size, these findings are reported as hypothesis‐generating effect size estimates to inform a future definitive trial, rather than as efficacy conclusions.

**TABLE 4:** Between‐group comparison of outcome variables across the study time points by generalised estimating equation modelling.

Outcomes	Time point	COMBAT‐ICU vs. attention control			Exercise‐only vs. attention control		
		Time*group interaction effect			Time*group interaction effect		
		*β* (95% CI)	*p*	Cohen's *d*	*β* (95% CI)	*p*	Cohen's *d*
PICSQ	T1	−1.97 (−3.54 to −0.40)	0.014	−0.50	−1.15 (−2.42 to 0.12)	0.341	−0.29
	T2	−1.76 (−3.46 to −0.05)	0.043	−0.45	−0.91 (−2.27 to 0.45)	0.497	−0.23
SPPB	T1	0.531 (−0.468–1.530)	0.297	0.28	0.621 (−0.584–1.826)	0.312	0.33
	T2	1.028 (−0.243–2.299)	0.113	0.54	0.765 (−0.460–1.991)	0.221	0.40
6MWT	T1	99.091 (−2.035–200.217)	0.055	0.93	94.924 (−9.054–198.903)	0.074	0.89
	T2	84.895 (−22.970–192.760)	0.123	0.79	59.318 (−26.842–145.478)	0.177	0.56
MoCA	T1	0.909 (−1.509–3.327)	0.461	0.26	−0.508 (−3.830–2.815)	0.765	−0.14
	T2	1.734 (−1.223–4.691)	0.250	0.49	−0.061 (−3.830–2.815)	0.970	−0.02
CTT‐1	T1	−9.220 (−21.275–2.834)	0.134	−0.45	−4.728 (−22.073–12.617)	0.593	−0.23
	T2	−12.202 (−29.182–4.778)	0.159	−0.60	−9.684 (−29.510–10.141)	0.338	−0.47
CTT‐2	T1	−20.743 (−58.153–16.667)	0.277	−0.46	−0.488 (−38.933–37.956)	0.980	−0.01
	T2	−8.957 (−42.759–24.845)	0.604	−0.20	5.532 (−40.447–51.511)	0.814	0.12
DST (F)	T1	0.755 (−0.067–1.577)	0.072	0.58	0.159 (−0.699–1.017)	0.716	0.12
	T2	0.944 (0.171–1.717)	0.017	0.73	0.553 (−0.277–1.383)	0.192	0.43
DST (B)	T1	0.420 (−0.291–1.130)	0.247	0.44	0.311 (−0.500–1.122)	0.453	0.32
	T2	0.196 (−0.713–1.104)	0.673	0.20	0.106 (−0.918–1.130)	0.839	0.11
GAD‐7	T1	−0.196 (−2.192–1.800)	0.848	−0.08	−0.189 (−2.769–2.390)	0.886	−0.07
	T2	−0.797 (−2.677–1.083)	0.406	−0.31	−0.432 (−2.944–2.080)	0.736	−0.17
PHQ‐9	T1	−0.755 (−2.798–1.287)	0.469	−0.29	−0.326 (−2.988–2.336)	0.810	−0.13
	T2	−0.839 (−2.667–0.989)	0.368	−0.32	−0.705 (−3.287–1.878)	0.593	−0.27
EQ‐5D‐5L index	T1	0.038 (−0.119–0.195)	0.634	0.29	0.000 (−0.169–0.168)	0.996	0.00
	T2	0.109 (−0.049–0.267)	0.175	0.84	0.067 (−0.088–0.221)	0.399	0.52
EQ‐5D‐5L (VAS)	T1	6.629 (−5.257–18.516)	0.274	0.53	5.924 (−4.442–16.290)	0.263	0.48
	T2	7.119 (−7.030–21.268)	0.324	0.57	7.439 (−5.048–19.927)	0.243	0.60

Abbreviations: 6MWT, 6‐min walk test; CCT, Colour Trail Test; DST (B), Digit Span Test (forward); DST, Digit Span Test (backward); EQ‐5D‐5L VAS, EuroQol 5‐Dimension 5‐Level (visual analogue scale); GAD‐7, Generalised Anxiety Disorder 7‐item scale; MoCA, Montreal Cognitive Assessment; PHQ‐9, Patient Health Questionnaire‐9; PICSQ, Post‐Intensive Care Syndrome questionnaire; SD, standard deviation; SPPB, Short Physical Performance Battery.

#### 4.4.1 PICS Severity

COMBAT‐ICU showed small‐to‐medium improvements versus attention control at both T1 (*d* = −0.50) and T2 (−0.45). The exercise‐only group showed smaller effects at both time points (T1: *d* = −0.29; T2: *d* = −0.23).

#### 4.4.2 Physical Function

Both active groups showed large between‐group effects for walking endurance (6MWT) versus attention control at T1 (COMBAT‐ICU: *d* = 0.93; exercise‐only: *d* = 0.89), attenuating to medium effects at T2 (COMBAT‐ICU: *d* = 0.79; exercise‐only: *d* = 0.56). For lower‐extremity performance (SPPB), effects were small at T1 for both groups, increasing to medium for COMBAT‐ICU (*d* = 0.54) and small‐to‐medium for exercise‐only (*d* = 0.40) at T2.

#### 4.4.3 Cognitive Function

COMBAT‐ICU consistently outperformed exercise‐only across cognitive domains. For global cognition (MoCA), COMBAT‐ICU showed a small effect at T1 (*d* = 0.26) increasing to upper‐medium at T2 (*d* = 0.49), while exercise‐only remained negligible at both time points. For attention and processing speed (CTT‐1), COMBAT‐ICU showed small‐to‐medium and medium effects at T1 and T2 (*d* = −0.45; *d* = −0.60), respectively. For short‐term memory (DST‐F), COMBAT‐ICU showed medium effects at both T1 (*d* = 0.58) and T2 (*d* = 0.73). Effects on higher‐order executive function (CTT‐2) and working memory (DST‐B) were small‐to‐negligible across both active groups.

#### 4.4.4 Mental Health

Between‐group effects on anxiety (GAD‐7) and depressive symptoms (PHQ‐9) were small across all active groups at both time points, suggesting that any additional benefit of either intervention on mental health beyond attention control is likely modest in this pilot sample.

#### 4.4.5 HRQoL

For the EQ‐5D‐5L index, COMBAT‐ICU showed a small effect at T1 (*d* = 0.29), increasing substantially to a large effect at T2 (*d* = 0.84), while exercise‐only showed a medium effect at T2 (*d* = 0.52). For self‐rated health (EQ‐5D‐5L VAS), both active groups showed medium effects at T1 and T2 (COMBAT‐ICU: *d* = 0.53–0.57; exercise‐only: *d* = 0.48–0.60).

## 5 Discussion

This pilot three‐arm RCT demonstrates that COMBAT‐ICU is feasible and safe for ICU survivors at risk of PICS. The absence of serious adverse events attributable to the interventions across all three groups confirms the safety profile of progressive home‐based exercise and cognitive training in this clinically vulnerable population. Exercise‐associated muscle soreness, an anticipated physiological response to progressive training, was the only exercise‐related symptom reported and did not result in session discontinuation or withdrawal. The 100% retention in COMBAT‐ICU and high session adherence rates (91.3% COMBAT‐ICU; 89.9% exercise‐only) strongly suggest that the intervention dose, frequency and delivery format were well tolerated.

The success of this blended home visit and online delivery model aligns with emerging evidence supporting remote rehabilitation for ICU survivors. Similar to the REACH pilot trial, which demonstrated high adherence and safety for an interdisciplinary home‐based program [26], COMBAT‐ICU successfully overcame common barriers to facility‐based programs, such as limited mobility and geographical constraints. Similarly, a systematic review of home‐based interventions for post‐ICU recovery reported that while findings were mixed or inconclusive regarding efficacy, home‐based delivery improved access for patients with limited mobility, social support or geographic barriers and reduced non‐attendance compared to facility‐based programs [27]. While a recent large‐scale iRehab trial tested a 6‐week remote multicomponent rehabilitation program across 52 hospitals and found no significant improvement in HRQoL at 8 weeks, secondary outcomes including leg strength, exercise capacity, fatigue and anxiety showed significant improvements [28]. The high adherence and robust engagement seen in COMBAT‐ICU suggest that its specific blend of structured contact and motor–cognitive integration is a viable model to test in a larger trial.

While this pilot was not powered to definitively evaluate efficacy, the exploratory clinical outcomes provide promising signals that justify a fully powered trial and offer important insights for its design. Physically, the overall trajectory of recovery in all groups was consistent with the natural functional gains typically seen in the overall trajectory of physical recovery, in which many ICU survivors experience spontaneous gains in functional capacity during the first months following discharge [29]. Exercise trials and systematic reviews have reported similar patterns, with significant within‐group increases in 6MWT distance and physical function in both intervention and attention control groups, but only small or inconsistent between‐group differences [6, 7]. A 2023 systematic review of nine RCTs evaluating post‐discharge rehabilitation programs confirmed this pattern: while both intervention and control groups showed improvements over time in functional capacity and 6MWT distance, most studies failed to demonstrate statistically significant between‐group differences, suggesting that accelerated recovery rather than absolute benefit may be the primary effect of structured rehabilitation [27]. Using the more sophisticated cardiopulmonary exercise test to objectively assess exercise capacity, McWilliams et al. (2016) similarly found no between‐group differences in peak VO_2_ or anaerobic threshold following a 7‐week outpatient program, despite significant improvements in HRQoL, illustrating the distinct trajectories of functional and psychological recovery [30]. In contrast, COMBAT‐ICU participants achieved gains substantially exceeding those reported in systematic reviews, suggesting the combined motor–cognitive program may augment recovery beyond the natural trajectory.

Exploratory cognitive outcomes further support the potential added value of structured cognitive training embedded within physical rehabilitation. COMBAT‐ICU demonstrated favourable between‐group trends for global cognition, attention, processing speed and short‐term memory relative to controls, aligning with evidence that combined physical‐cognitive interventions outperform exercise alone [31, 32, 33, 34]. However, the limited effect on higher‐order executive function provides a critical lesson for a definitive trial. This limitation likely reflects the sequential nature of the intervention (cognitive training following exercise); meta‐analytic evidence indicates that executive gains require simultaneous cognitive‐motor interference [35]. Consequently, incorporating simultaneous dual‐task paradigms will be a logical refinement for future trial design.

Finally, exploratory findings regarding mental health and HRQoL mirror the cautiously favourable literature on post‐ICU interventions [36, 37]. Although the brief intervention yielded only small between‐group effects for depression and anxiety, likely due to the high rate of spontaneous psychological improvement in the early post‐ICU period, the favourable effect sizes for EQ‐5D‐5L index scores are noteworthy. Because the EQ‐5D‐5L captures the integrated functional and psychological experience of health, these preliminary patterns suggest that a multidimensional, home‐based program may confer broader psychosocial benefits than exercise alone. Collectively, these exploratory efficacy signals confirm that the COMBAT‐ICU model warrants a large‐scale, definitive RCT with longer follow‐up to substantiate these preliminary psychosocial and functional gains.

### 5.1 Limitations

This pilot study has several methodological limitations. First, the small sample size inherently restricts statistical power, increasing the risk of Type II errors [38]; consequently, several clinically meaningful effect estimates did not achieve statistical significance, and the wide confidence intervals reported throughout should be interpreted as reflecting estimation uncertainty rather than an absence of effect. The sample size was determined in accordance with established guidance for pilot feasibility studies [24], which recommend this as adequate to assess feasibility outcomes and generate preliminary effect size estimates for future definitive trials. However, this limited sample also precluded subgroup analyses (e.g., by age or ICU diagnosis) and cannot entirely rule out residual confounding despite randomisation. Second, the three‐month post‐intervention follow‐up is insufficient to capture longer‐term recovery trajectories or the potential late emergence of PICS manifestations. Finally, requiring participants to have internet access and live with family restricts the generalisability of these findings, particularly concerning socially isolated survivors, those in resource‐limited settings or differing healthcare systems. Future fully powered RCT with extended longitudinal follow‐ups and broader inclusion criteria are warranted to validate these findings and evaluate long‐term efficacy across diverse populations.

### 5.2 Implications for Practice and/or Further Research

The findings of this pilot trial suggest that a home‐based, blended motor–cognitive program for ICU survivors at risk of PICS is clinically feasible and may offer multidomain benefits beyond those of exercise alone. Where resources permit, these results support the integration of structured cognitive training into post‐ICU rehabilitation pathways. To definitively evaluate the efficacy of COMBAT‐ICU, a fully powered, two‐arm RCT is warranted. Based on the conservative between‐group effect size for PICS severity observed at T2 (Cohen's *d* = 0.45) and the highest arm‐level attrition rate (18%) documented in this pilot, a future trial would require 156 completers to achieve 80% power at *α* = 0.05 (two‐sided). Accounting for a 20% attrition buffer to ensure adequate power retention, a total randomised sample size of 196 participants (98 per arm) would be necessary. This sample size is feasible for a multicentre trial conducted over 24 months of recruitment, with corresponding implications for intervention delivery resources, staff training and long‐term follow‐up infrastructure.

## 6 Conclusion

This pilot three‐arm RCT demonstrates that COMBAT‐ICU is feasible, safe and acceptable, with high adherence and retention. As a secondary exploratory aim, preliminary findings suggest broader improvements across physical, cognitive and HRQoL domains compared with exercise alone, although effects on higher‐order executive function were limited. These results support progression to a fully powered multicentre trial incorporating explicit dual‐task executive training, extended follow‐up and broader inclusion criteria to confirm efficacy and optimise delivery.

## Funding

This study was supported by the University of Hong Kong, Seed Fund for PI Research—Basic Research, Grant No. 109001164.

## Ethics Statement

This study was approved by the Institutional Review Board of the University of Hong Kong/Hospital Authority Hong Kong West Cluster, Reference No. UW‐23‐271 and Central Institutional Review Board of the Hospital Authority Hong Kong, Reference No. CIRB‐2023‐087‐2.

## Consent

Written informed consent was obtained from all participants prior to enrolment in the study.

## Conflicts of Interest

The authors declare no conflicts of interest.

## Data Availability

The data that support the findings of this study are available on request from the corresponding author. The data are not publicly available due to privacy or ethical restrictions.
